# Investigation of a measles outbreak in Brondong subdistrict, Lamongan district, Indonesia, 2023

**DOI:** 10.5365/wpsar.2025.16.1145

**Published:** 2025-11-03

**Authors:** Konstantinus Ua, Lucia Yovita Hendrati, Kornelius Langga Son, Siti Shofiya Novita Sari, Erni Astutik, Sigunawan

**Affiliations:** a Indonesia Field Epidemiology Training Program, Universitas Airlangga, Surabaya, Indonesia.; b Epidemiology Division, Department of Epidemiology, Biostatistics, Population and Health Promotion, Faculty of Public Health, Universitas Airlangga, Surabaya, Indonesia.; c Indonesia Epidemiological Association, Jakarta, Indonesia.; d Lamongan District Health Office, East Java Province, Indonesia.

## Abstract

**Objective:**

Lamongan District Health Office received a report of a suspected measles outbreak from a community health centre and two hospitals in Brondong subdistrict, Lamongan district, Indonesia. An outbreak investigation team was deployed to verify the diagnosis and to determine the magnitude of the outbreak.

**Methods:**

This retrospective, 1:1 matched case-control study involved 51 suspected or laboratory-confirmed measles cases and 51 controls selected from household contacts and/or playmates within the same village who did not have measles. Data on case characteristics, clinical symptoms, vaccination status, as well as contact and travel histories were collected via interview. Blood specimens were collected from 25 of the children for laboratory confirmation. Univariate and multivariable logistic regression analyses were conducted to investigate risk factors for measles infection.

**Results:**

Nineteen of the 51 measles cases (37.3%) were laboratory-confirmed. All 51 cases exhibited fever and rash (100%) and ranged in age from 11 months to 12 years; 29 (56.9%) were female, and 32 (62.7%) were hospitalized. Over half of the cases occurred in Sedayulawas village (31/51, 60.8%), showing a propagated epidemic pattern. The index case was a 2-year-old girl. Transmission predominantly occurred within the same village through household or playmate contacts. Immunization status and contact history were significantly associated with measles infection.

**Discussion:**

The measles outbreak was attributed to a decline in immunization coverage, particularly for the second dose of the measles-rubella vaccine. This decrease was driven by multiple factors, including the impact of the COVID-19 pandemic, misconceptions related to religious beliefs, and the long interval between the first and second vaccine doses, which contributed to the patients who were lost to follow-up. Collectively, these factors increased the vulnerability of children to measles infection.

Measles is a highly contagious disease, spread by contact with infected nasal or throat fluids (via coughing or sneezing) or by inhaling air exhaled by a person with measles. The virus remains active and contagious in the air or on infected surfaces for up to 2 hours. (*1*) Measles infection can lead to severe illness, complications and even death. (*1*) Children aged < 5 years face heightened risks of severe complications, with 1–3 out of every 1000 infected children succumbing to respiratory and neurological issues. (*2*)

Despite the commitment of the Government of Indonesia to eliminate measles and rubella by 2023, cases of measles have increased in recent years. In 2022, a total of 55 measles outbreaks were reported in 12 of the country’s 38 provinces and, during the first quarter of 2023, outbreaks were reported in 18 provinces. Indonesia’s elimination strategy has focused on achieving uniform 95% measles and rubella vaccine (MR) coverage through immunization campaigns and programme integration. (*3*) However, according to United Nations Children’s Fund (UNICEF) and World Health Organization (WHO) estimations, the persistently low MR immunization coverages of below 95% over the past 3 years (2020–2022), which was further exacerbated by the COVID-19 pandemic, left approximately 0.8 million and 0.6 million children undervaccinated for MR1 and MR2, respectively, (*4*, *5*) thereby jeopardizing the 2023 elimination goal.

Between 1 January and 3 April 2023 alone, a total of 2161 measles cases (848 laboratory-confirmed and 1313 clinically suspected) were reported across Indonesia. One of the affected provinces was East Java, which includes Lamongan district. (*6*) By the end of 2023, Lamongan district had recorded a total of 253 suspected and confirmed cases across its 18 subdistricts, a significant increase over the 22 recorded cases in 2022. (*7*)

In early July 2023, Lamongan’s district health office (DHO) was notified of six children diagnosed with clinical measles, with symptoms of fever, rash, cough and conjunctivitis, who were treated at either a community health centre or one of two hospitals. (*8*) This report describes the results of a subsequent comprehensive epidemiological investigation of the measles outbreak in the two affected villages, Brondong and Sedayulawas, in Brondong subdistrict, Lamongan, East Java.

## Methods

### Study design and population

A 1:1 matched case-control study design was used as the basis of the investigation. Suspected and confirmed measles cases were identified from Lamongan DHO reports for June and July 2023. Case definitions were based on WHO definitions of suspected and confirmed measles cases: (*9*)

Suspected case: fever and generalized maculopapular (non-vesicular) rash with at least one of the following: cough, coryza or conjunctivitis, in any child aged 0–12 years presenting or residing in Brondong and Sedayulawas villages between June and July 2023.
Confirmed case: any suspected case with measles IgM antibodies between June and July 2023.

Matched controls were recruited from the pool of children (aged 0–12 years) who had none of the above-mentioned symptoms and resided in the same household as the cases or were playmates (that is, had contact with a case during June–July 2023).

### Data collection

Face-to-face interviews with parents, using a standard questionnaire developed by the Ministry of Health, were conducted to collect data on participants’ characteristics, including age, sex, symptoms, rash onset, home address, health-care facilities visited, immunization history, contact history with measles cases within 2 weeks, measles history and travel history. Secondary data sources were also used, including surveillance data, medical records, laboratory results from specimen testing, population and coverage data on MR immunization; these data were obtained from community health centres, local hospitals, the referral laboratory and the Lamongan DHO.

### Data analysis

Descriptive analysis was used to describe the characteristics of the study cohort. Univariate analysis using the χ^2^ test was performed to calculate crude odds ratios (cORs), 95% confidence intervals (CIs) and *P*-values for the association between potential risk factors and measles infection. Risk factor variables that were significant at the level of *P* < 0.25 in univariate analysis were selected for inclusion in a multivariable logistic regression analysis. For adjusted odds ratios (aORs), two-sided *P*-values of < 0.05 were considered to indicate significance, and 95% CIs were calculated. Variables included in the multivariable analysis were sex, age group, immunization status, contact history and travel history.

All statistical analyses were performed using STATA version 16.

## Results

### Descriptive epidemiology

A total of 51 measles cases were identified during the study period. Of the 25 serum samples collected, 19 were positive for measles IgM. The age of cases ranged from 11 months to 12 years (median 5 years). The age group with the highest age-specific attack rate was children aged 5–9 years (24, 47.1%), followed by those aged < 5 years (21, 41.2%). Females accounted for 29 cases (56.9%). Thirty-two cases (62.7%) were hospitalized, while the remaining 19 cases (37.3%) sought outpatient treatment at a health centre. In addition to fever and rash, most cases had cough (96.1%) and nearly half had conjunctivitis (45.1%). Over half of cases were from Sedayulawas village (31, 60.8%). Only three of the 51 cases (5.9%) had received at least one dose of measles vaccine. In terms of contact history, 35 cases (68.6%) had contact history with measles cases within 2 weeks of rash onset, while 18 cases (35.3%) had no record of travelling to areas with measles outbreak status (**Table 1**).

**Table 1:** Characteristics of measles cases and controls, Brondong and Sedayulawas villages, Lamongan district, East Java, Indonesia, 2023 (*n* = 102)

Characteristic	Cases (*n* = 51)		Controls (*n* = 51)	
	*n*	%	*n*	%
**Sex**				
**Male**	**22**	**43.1**	**29**	**56.9**
**Female**	**29**	**56.9**	**22**	**43.1**
**Age group, years**				
**0–4**	**21**	**41.2**	**10**	**19.6**
**5–9**	**24**	**47.1**	**30**	**58.8**
**10–14**	**6**	**11.8**	**11**	**21.6**
**Symptoms**				
**Fever**	**51**	**100.0**	**NA**	**NA**
**Rash**	**51**	**100.0**	**NA**	**NA**
**Cough**	**49**	**96.1**	**NA**	**NA**
**Cold**	**9**	**17.6**	**NA**	**NA**
**Red eyes**	**23**	**45.1**	**NA**	**NA**
**Diarrhoea**	**4**	**7.8**	**NA**	**NA**
**Other**	**5**	**9.8**	**NA**	**NA**
**Place of residence**				
**Sedayulawas village**	**31**	**60.8**	**34**	**66.7**
**Brondong village**	**20**	**39.2**	**17**	**33.3**
**Case type**				
**Suspected**	**26**	**51.0**	**NA**	**NA**
**Laboratory confirmation (+)**	**19**	**37.3**	**NA**	**NA**
**Laboratory test (-)**	**6**	**11.8**	**NA**	**NA**
**Place of care**				
**Hospital**	**32**	**62.7**	**NA**	**NA**
**Health centre**	**19**	**37.3**	**NA**	**NA**
**MR immunization status**				
**Received one or two doses**	**3**	**5.9**	**11**	**21.6**
**Not immunized**	**48**	**94.1**	**40**	**78.4**
**Contact history**				
**Yes**	**35**	**68.6**	**22**	**43.1**
**No**	**16**	**31.4**	**29**	**56.9**
**Travel history**				
**Yes**	**33**	**64.7**	**21**	**41.2**
**No**	**18**	**35.3**	**30**	**58.8**

MR: measles-rubella vaccine; NA: not applicable.

**Fig. 1:**
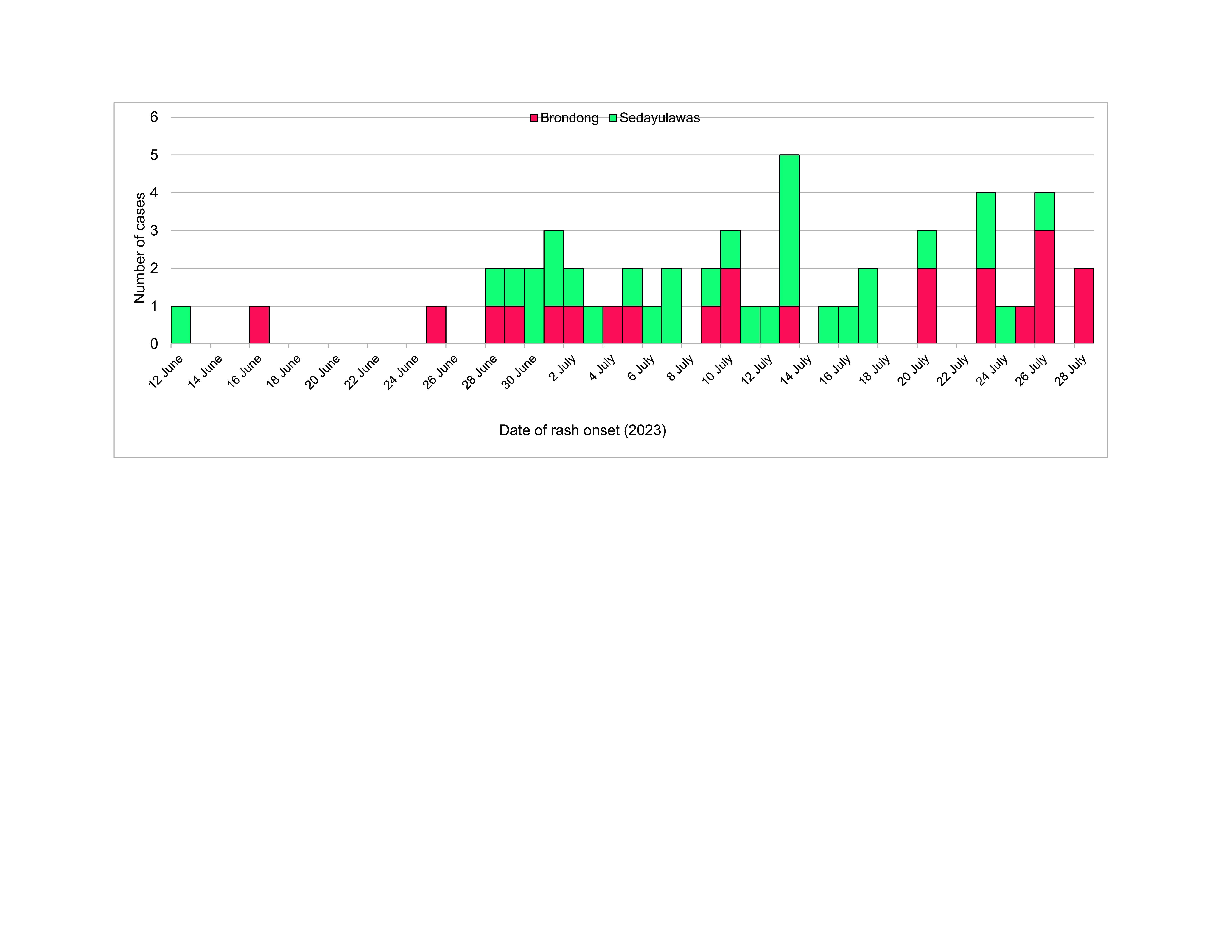
Epidemic curve of a measles outbreak in Brondong and Sedayulawas villages, Lamongan district, East Java, Indonesia, 2023 (^N^ = 51)

The earliest date of rash onset among the 51 measles cases was 12 June 2023. Cases steadily increased from 1 July onwards, peaked around 13 July, and ended on 28 July (**Fig. 1**). The index case was a 2-year-old girl from Sedayulawas village.

[**insert** Figure 1]

### Measles risk factors

Multivariable analysis indicated that only immunization status and contact history were significantly associated (*P* < 0.05) with measles infection. Not being immunized increased the odds of measles more than 4-fold (aOR: 4.7, 95% CI: 1.1–20.6) relative to being immunized. Contact with a measles case in the preceding 2 weeks increased the odds of measles by a factor of 3.6 (aOR: 3.6, 95% CI: 1.4–8.9) relative to no contact (**Table 2**).

**Table 2:** Logistic regression analysis of factors associated with measles outbreak, Brondong, Lamongan, East Java, Indonesia, 2023

Risk factor	Measles status		Crude odds ratio (95% CI)	Adjusted odds ratio (95% CI)
	Cases	Controls		
**Sex**				
**Male**	**22**	**29**	**Ref**	**Ref**
**Female**	**29**	**22**	**1.7 (0.7–4.1)**	**1.9 (0.8–4.7)**
**Age group, years**				
**0–4**	**21**	**10**	**3.85 (0.1–39.6)**	**2.3 (0.5–8.8)**
**5–9**	**24**	**30**	**0.9 (0.1–14.5)**	**1.0 (0.3–3.5)**
**10–14**	**6**	**11**	**Ref**	**Ref**
**Immunization status**				
**Received one or two doses**	**3**	**11**	**Ref**	**Ref**
**Not immunized**	**48**	**40**	**4.4 (1.1–25.8)**	**4.7 (1.1–20.6)**
**Contact history**				
**No**	**16**	**29**	**Ref**	**Ref**
**Yes**	**35**	**22**	**2.9 (1.2–7.0)**	**3.6 (1.4–8.9)**
**Travel history**				
**No**	**18**	**30**	**Ref**	**Ref**
**Yes**	**33**	**21**	**2.6 (1.1–6.3)**	**2.3 (0.9–5.5)**

Bold values are statistically significant (*P* < 0.05).

### Vaccination coverage

In Lamongan district, average coverage of MR1 (at 9 months) declined from 95.1% in 2019 to 86.2% during the COVID-19 pandemic, while coverage of MR2 (at 18 months) fell from a pre-pandemic level of 73.1% to 65.4%. (*8*) Brondong village experienced a similar drop in MR1 coverage, by 12.2% from 100.7% in 2020 to 88.5% in 2022. However, relative to the district average, MR2 coverage rates were much lower, only reaching 8.7%, 6.4% and 10.1% in 2020, 2021 and 2022, respectively. The situation was similar in Sedayulawas village, where MR1 coverage decreased by 8.8% from 101.1% to 92.3% over the same 3-year period, with coverage below 95% in both 2021 and 2022. MR2 coverage also remained low, at less than 10% between 2020 and 2022 (8.2%, 6.1% and 6.3%, respectively). (*7*)

## Discussion

Despite Indonesia’s efforts to eliminate measles by 2023, new cases continue to emerge in various regions, including Lamongan district. Outbreaks have occurred in Brondong and Sedayulawas villages, where there is a high proportion of unvaccinated children. Vaccination coverage data for the district as a whole and for the affected villages show marked declines in MR vaccine coverage from pre-pandemic levels, especially for MR2. Studies conducted in other countries have shown that low MR vaccine coverage and weak immunization delivery systems can lead to the buildup of groups of children susceptible to measles, increasing the risk of outbreaks. (*10*-*12*)

This study showed a strong association between vaccination history and measles infection and underscores the importance of maintaining MR immunization coverage levels above 95%, particularly among children aged < 5 years who are most vulnerable to the effects of measles. This effort is essential in establishing herd immunity and preventing measles outbreaks. (*11*, *12*)

This study also showed an association between contact history and measles infection, which is consistent with that of other studies, including one conducted in Ethiopia. (*13*) In contrast, we found no association between travel history and measles infection. This suggests that transmission occurred predominantly within the Brondong subdistrict, presumably via household contacts, with few imported cases. A previous outbreak investigation conducted in two villages in Jiken subdistrict, Blora Regency, reached a similar conclusion. (*14*)

The recent COVID-19 pandemic has undoubtedly contributed to the recent decline in MR vaccination coverage and increase in measles cases in many parts of Indonesia. However, there are also likely other underlying factors that have contributed to low MR vaccine coverage, especially of MR2, that are more unique to Indonesia. These include parents or caregivers refusing to vaccinate their children due to religious beliefs (concerns that vaccines are not halal or are haram) and parental concerns about the incidence of adverse events following immunization. The high drop-out rate between the first and second dose is particularly concerning. Anecdotal evidence suggests that this may be due in part to a belief that one dose is sufficient, and the second dose is not necessary. Another reason might be recent changes to the MR2 immunization schedule. The latter has been cited as a factor in an Ethiopian study where changes to the measles vaccine schedule, which were more inconvenient for caregivers, were ranked alongside displacement as one of the most frequently cited reasons for not bringing children to clinics for their second measles vaccination (24.1%). (*15*) This was closely followed by misunderstandings about immunization. This study also reported very high drop-out rates among Muslim study participants (77.1%).

Our study had several limitations. Controls were limited to household contacts and playmates within the same villages. In addition, some cases may have been missed due to time constraints and limited opportunities for specimen collection. The immunization status of respondents was based on verbal reports. Most participants did not have vaccination cards or proof of vaccination, so recall bias was unavoidable. Despite these limitations, this outbreak investigation confirmed the pattern of measles infection in young children (0–9 years) and the clustering of measles cases in the same village.

Based on this investigation, it is recommended that outbreak response immunization be conducted to prevent measles transmission in the affected areas. In addition, MR1 and MR2 immunization coverage should be increased in those areas and districts where coverage is currently below the national target (< 95%). In addition, a strategy is needed to reduce MR2 dropout by conducting home visits and improving the provision of MR vaccines through better coordination between health services and the community. At the national level, cross-sectoral coordination should be improved to increase and maintain vaccination coverage across Indonesia. In addition, health promotion should be conducted to increase public understanding and awareness of measles immunization, and the early detection and monitoring of suspected measles cases should be conducted through active and passive surveillance.

## References

[1] Measles [website]. Geneva: World Health Organization; 2023. Available from: https://www.who.int/news-room/fact-sheets/detail/measles, accessed 9 December 2023.

[2] Clinical overview of measles [website]. Atlanta (GA): United States Centers for Disease Control and Prevention; 2025. Available from: https://www.cdc.gov/measles/hcp/clinical-overview/index.html, accessed 10 September 2025.

[3] Kemkes RI. [Measles rubella surveillance guidelines]. Vol. I. Jakarta: Directorate General of Disease Control; 2023 (in Indonesian). Available from: https://sites.google.com/view/panduansurveilans/kumpulan-pedoman/campak, accessed 15 April 2024.

[4] WHO/UNICEF estimates of national immunization coverage: 2024 revision [website]. Geneva and New York (NY): World Health Organization/United Nations Children’s Fund; 2024. Available from: https://worldhealthorg.shinyapps.io/wuenic-trends/, accessed 15 September 2025.

[5] Immunization country profiles [website]. New York (NY): United Nations Children’s Fund (UNICEF); 2025. Available from: https://data.unicef.org/resources/immunization-country-profiles/, accessed 27 August 2025.

[6] Disease outbreak news: measles – Indonesia [website]. Geneva: World Health Organization; 2023. Available from: https://www.who.int/emergencies/disease-outbreak-news/item/2023-DON462, accessed 15 April 2024.

[7] Immunization – East Java Provincial Health Office [website]. Surabaya: East Java Provincial Health Office; 2022 (in Indonesian). Available from: https://imun.aksi.web.id, accessed 15 April 2024.

[8] Lamongan district health profile [website]. Lamongan: Lamongan District Health Office; 2022 (in Indonesian). Available from: https://lamongankab.go.id/beranda/dinkes/post/1872, accessed 15 April 2024.

[9] Measles outbreak guide. Geneva: World Health Organization; 2022. Available from: https://iris.who.int/handle/10665/360891, accessed 15 April 2024.

[10] Dzeyie KA, Lowang D, Dikid T, Wangsu W, Tamir T; Working Group*. Measles outbreak investigation at Indo-Myanmar border, Longding District, Arunachal Pradesh, India, 2017. Indian J Public Health. 2021 Jan;65(5) Supplement:S23–8. 10.4103/ijph.IJPH_1067_20 33753588

[11] Oktaviasari KE. Relationship of measles immunization with measles in East Java. Jurnal Berkala Epidemiologi. 2018;6(2):166–73. 10.20473/jbe.V6I22018.166-173

[12] Al Machmudi MI. [Increase in measles cases due to declining immunization coverage] [website]. Media Indonesia; 2023 (in Indonesian). Available from: https://mediaindonesia.com/humaniora/551790/peningkatan-kasus-campak-karena-menurunnya-angka-imunisasi, accessed 17 August 2023.

[13] Girmay A, Dadi AF. Being unvaccinated and having a contact history increased the risk of measles infection during an outbreak: a finding from measles outbreak investigation in rural district of Ethiopia. BMC Infect Dis. 2019 Apr 25;19(1):345. 10.1186/s12879-019-3973-8 31023269 PMC6485078

[14] Napitupulu D, Kolawi AP, Pramono D, Mualim K. [Household contact as a factor in measles outbreaks in two villages of Jiken Blora subdistrict]. Berita Kedokteran Masyarakat. 2018;34(5):1–5 (in Indonesian). doi:10.22146/bkm.37618

[15] Hailu C, Fisseha G, Gebreyesus A. Determinants of measles vaccination dropout among 12 - 23 months aged children in pastoralist community of Afar, Ethiopia. BMC Infect Dis. 2022 Apr 14;22(1):376. 10.1186/s12879-022-07350-1 35421952 PMC9008940

